# Array comparative genomic hybridization analyses of all blastomeres of a cohort of embryos from young IVF patients revealed significant contribution of mitotic errors to embryo mosaicism at the cleavage stage

**DOI:** 10.1186/1477-7827-12-105

**Published:** 2014-11-24

**Authors:** Judy FC Chow, William SB Yeung, Estella YL Lau, Vivian CY Lee, Ernest HY Ng, Pak-Chung Ho

**Affiliations:** Department of Obstetrics and Gynaecology, The University of Hong Kong, Queen Mary Hospital, Pokfulam, Hong Kong; Department of Obstetrics and Gynaecology, Queen Mary Hospital, Pokfulam, Hong Kong

**Keywords:** Cleavage stage embryo, Mosaicism, Aneuploidy

## Abstract

**Background:**

Embryos produced by in vitro fertilization (IVF) have a high level of aneuploidy, which is believed to be a major factor affecting the success of human assisted reproduction treatment. The aneuploidy rate of cleavage stage embryos based on 1–2 biopsied blastomeres has been well-reported, however, the true aneuploidy rate of whole embryos remain unclear because of embryo mosaicism. To study the prevalence of mosaicism in top quality IVF embryos, surplus embryos donated from young patients (aged 28–32) in the assisted reproduction program at Queen Mary Hospital, Hong Kong were used.

**Methods:**

Thirty-six good quality day 2 embryos were thawed. Out of the 135 blastomeres in these embryos, 121 (89.6%) survived thawing. Twelve of these embryos without lysed blastomeres and which cleaved to at least seven cells after a 24-h culture were dissembled into individual blastomeres, which were analysed by array comparative genomic hybridization and microsatellite marker analysis by fluorescent PCR.

**Results:**

Out of 12 day-3 embryos, 2 (16.7%) were normal, 3 (25%) were diploid/aneuploidy with <38% abnormality, 4 (33.3%) were diploid/aneuploidy mosaic with > =38% abnormality, and three (25%) were mosaic aneuploids. Conclusive chromosomal data were obtained from a high percentage of blastomeres (92.8%, 90/97). Microsatellite marker analysis performed on blastomeres in aneuploid embryos enabled us to reconstruct the chromosomal status of the blastomeres in each cleavage division. The results showed the occurrence of meiotic errors in 3 (25%) of the studied embryos. There were 16 mitotic errors (18.8%, 16/85) in the 85 mitotic divisions undertaken by the studied embryos. The observed mitotic errors were mainly contributed by endoreduplication (31.3%, 5/16), non-disjunction (25%, 4/16) and anaphase lagging (25%, 4/16). Chromosome breakages occurred in 6 divisions (7.1%, 6/85).

**Conclusions:**

Mosaicism occurs in a high percentage of good-quality cleavage stage embryos and mitotic errors contribute significantly to the abnormality.

**Electronic supplementary material:**

The online version of this article (doi:10.1186/1477-7827-12-105) contains supplementary material, which is available to authorized users.

## Background

Embryos derived from in vitro fertilization have a high level of aneuploidy [1, 2], which is believed to be a major factor affecting success of human assisted reproduction treatment. Therefore, preimplantation genetic screening (PGS), i.e., screening for chromosomal anomalies in preimplantation embryos, is advised before embryo transfer in treatment. Previously, PGS was performed using fluorescence in situ hybridization (FISH) on 5–12 chromosomes. Subsequent randomized controlled trials failed to show a benefit of PGS by FISH on the outcome of assisted reproduction, due to embryo mosaicism and technical limitation of FISH in analysing only a limited number of chromosomes [3, 4]. In recent years, array-based comparative chromosomal hybridization (aCGH) was developed to solve the latter problem. With this method, all 24 chromosomes in a single cell can be analysed. Two recent randomized trials show an improvement in success rate after PGS by aCGH [5] and quantitative polymerase chain reaction (PCR) based aneuploidy screening [6].

Although mosaicism and aneuploidy in cleavage stage embryos based on 1–2 biopsied blastomeres have been well reported, the true aneuploidy rate and the extent of mosaicism are not as clear as it requires analysis of all 24 chromosomes in every blastomeres in an embryo. There are only a few studies reporting such information on a limited number of cleavage stage embryos. Two studies used abnormal embryos derived from preimplantation genetic diagnosis (PGD) or PGS programs [7, 8]. Several reports used cryopreserved embryos with quality suitable for transfer [9–11]. However, due to local regulatory requirements, the investigators of these reports allowed the embryos to succumb at room temperature for 24 h before determination of the chromosomal content [10, 11]. The effect of the treatment on chromosome separation is not known. Notably, a significant proportion of the blastomeres in these studies failed to produce a conclusive result. Malmgren et al. [12] studied 22 embryos unsuitable for transfer after PGD for structural aberration and 6 embryos donated from IVF patients with no known structural aberration. These embryos had a total cell number from 1–10 on day 4 of culture when their chromosomal content was determined by CGH. Wells and Delhanty [13] studied 12 embryos on day 3 of culture with some having cleavage arrest. Johnson and co-workers [14] studied 26 day 3 cryopreserved embryos from a group of advanced aged women with a mean age of 38.8. There is no data on mosaicism of high quality embryos from young women without chromosomal abnormality.

Both meiotic errors and post-zygotic mitotic errors contribute to aneuploidy in preimplantation embryos. The reported mechanisms causing these errors include non-disjunction, anaphase lagging and selective endoreduplication. Non-disjunction produces two daughter cells with one gain and one loss of the same chromosome. It results from failure to properly separate the sister chromatids during mitosis. Anaphase lagging is due to failure in attachment of a chromatid to the spindle apparatus and subsequent exclusion from the reforming nucleus. Selective endoreduplication refers to the replication of a chromosome without cell division, resulting in one normal cell and one with trisomy of the reduplicated chromosome. It has been suggested that anaphase lag leading to chromosome loss is the most common mechanism causing mosaicism [15].

Most data on aneuploidy in preimplantation embryos were derived from studies using FISH for determination of the chromosomal content. These studies had three shortcomings. First, most of the studied embryos were diagnosed to be abnormal after PGS. Second, only a limited number of chromosomes were studied. Third, not all blastomeres of the embryos were investigated.

In this report, we aim to determine the extent of mosaicism in good quality frozen embryos from young IVF patients with no known indication for PGD. The chromosome content of each individual blastomere was analysed by aCGH, and microsatellite marker analysis were performed on the aneuploid chromosomes.

## Methods

### Embryos

The study was approved by the Institutional Review Board of the University of Hong Kong/Hospital Authority Hong Kong West Cluster (IRB reference number: UW 13–019) and the Council on Human Reproductive Technology, Hong Kong (research licence no. R3002). The embryos used in this research were donated from patients in the assisted reproduction program at Queen Mary Hospital, Hong Kong. Signed consents were obtained from all of the donors. The embryos were fertilized by intracytoplasmic sperm injection, cultured in 12 μl microdroplets of G-1 medium (Vitrolife AB, Göteborg, Sweden) for approximately 48 h in a humidified atmosphere of 6% carbon dioxide, 5% oxygen and 89% nitrogen. Cleavages of the embryos were examined every 24 h until they were cryopreserved with slow freezing on day 2 of culture. Between September 2013 and January 2014, the embryos were thawed and cultured for 24 h before individual blastomeres were collected by biopsy needle under an inverted microscope. To ensure that only good quality embryos were studied, we selected the studied embryos by three criteria. First, they were 3-4-celled with less than 25% fragmentation at the time of cryopreservation. Second, all of their blastomeres survived the thawing procedure. Third, the embryos cleaved to at least 7-cells upon in vitro culture for 24 h.

### Array based comparative genomic hybridization

Each biopsied blastomere was washed in three 5 μl droplets of sterile 1.5% polyvinylpyrrolidone in phosphate buffered saline in a laminar flow hood and then placed in a 0.2 μl PCR tube with a minimal volume of medium. They were stored at -80 °C until experimentation. Whole genome amplification was performed by SurePlex DNA amplification system (BlueGnome, Cambridge, UK) according to the manufacturer’s protocol. The final wash medium was used as a negative control and 3 ng of normal female genomic DNA as a positive control. aCGH was performed using 24 sure v3 slides (BlueGnome). After scanning of the microarray slides, the images were analysed using BlueFuse Multi software (v 3.1). All genomic positions refer to the human genome build NCBI 37.

### Analyses on microsatellite markers

Each blastomere in an abnormal embryo was analysed by haplotyping approach [16]. In brief, Sureplex amplified DNA of the blastomere was purified with the Qiaquick PCR purification system (Qiagen, Manchester, UK). Multiplex fluorescent PCR was performed on three to seven microsatellite markers of the aneuploid chromosome (chromosomes 2, 11, 14, 15, 16, 19 or 20). PCR products were separated using a ABI 3500 Genetic Analyzer (Applied Biosystems, Foster City, USA) and the data were analysed using GeneMapper v4.1 (Applied Biosystems).

### Reconstruction of cell divisions

All embryos were examined daily after fertilization. Based on the size and number of blastomeres observed every 24 h, no trichotomic mitosis was observed. Trichotomic mitosis refers to either zygotes cleaved into 3 blastomeres or 2-cell embryos into 5–6 cells. We analysed the 24 chromosomes in each blastomere using aCGH. Microsatellite analysis was performed on the aneuploid chromosomes of the mosaic embryos. These analyses were used to distinguish trisomy due to meiotic error (three alleles detected), endoreduplication (two alleles detected) and uniparental disomy (one allele detected). Based on the results aCGH and microsatellite marker analysis, we reconstructed the chromosomal content of cell lineages of each embryo, assuming 1) events leading to chromosomal aberration were by reported mechanisms including anaphase lagging, non-disjunction, selective endoreduplication and chromosome breakage; 2) the minimal number of events that could explain the chromosomal status of the day 3 embryos studied; and 3) one event per cell division as far as possible.

## Results

### Patient demography

Twelve embryos included in this study were donated by four couples. These couples underwent assisted reproduction treatment with intracytoplasmic sperm injection treatment for severe male factor. Three men had oligoasthenoteratozoospermia with very low sperm counts (<2 × 10^6^/ml), low motility (<50%) and low normal morphology (<30%), according to WHO (1999) guidelines. One man had only abnormal sperm morphology (<10%) with normal sperm count and low motility (<50%) All patients had a history of infertility ranging from 3 to 8 years. The mean age of the wife and husband was 30 (range 28–32) and 37 (range 30–45), respectively. The number of embryos donated from each patient ranged from 2–4. Seven of the 12 embryos (58.3%) were from successful treatment cycles resulting in live births.

### Embryo development after culture

Altogether, 36 embryos were thawed, representing 135 blastomeres, of which 121 survived upon thawing. The survival rate of blastomeres after thawing was 89.6% which was similar to that of the frozen-thawed embryo transfer in our IVF program. Twelve of these embryos without lysed blastomeres and which cleaved to at least seven cells after a 24-h culture were dissembled and analysed by aCGH. The morphological grading of the 12 thawed embryos after culture is shown in Table 1. The average number of blastomeres per embryo on day 3 was 7.8 ± 0.6 (range 7–10). Seven of them were grade 1 (equal sized blastomeres without fragmentation) and grade 2 (equal sized blastomeres with <25% fragmentation), two were grade 3 (unequal size blastomeres without fragmentation), and three were grade 4 (unequal size blastomeres with <25% fragmentation).

**Table 1:** **aCGH result of analysed blastomeres on day 3 embryos**

Embryo	Patient	Age	Day 3 cell number (grade)	Number of cells studied	Genotype (no. of cell)	Category
1	B	31	8(2)	8	46,XX (7);	Diploid - aneuploid mosaic (<38%)
					49,XX,+2,+13,+14 (1)	
2	B	31	8(1)	8	46,XY (8)	Normal
3	C	32	8(1)	8	46,XX (4)	Diploid - aneuploid mosaic (>38%)
					44,XX,-14,-15 (1)	
					48,XX,+14,+15 (1)	
					46,XX,del(Xq21.2-qter) (1)	
					No result (1)	
4	C	32	8(1)	8	43XY,-14, -15,-16 (1)	Aneuploid mosaic
					47,XY,+14,+15,-16 (2)	
					45,XY,-16 (4)	
					No result (1)	
5	C	32	7(4)	7	45,XX,-20 (4)	Aneuploid mosaic
					44,XX,-4,-20 (1)	
					No result (2)	
6	D	28	8(2)	8	46,XX,del(2pter-p16.3) (6)	Aneuploid mosaic
					46,XX,dup(2pter-p16.3) (2)	
7	D	28	8(3)	8	46,XY (2)	Diploid – aneuploid mosaic (>38%)
					47,XY,+16 (5)	
					Chaotic (1)	
8	D	28	9(3)	9	46,XX (6)	Diploid – aneuploid mosaic (<38%)
					47,XX,+19 (2)	
					Chaotic (1)	
9	E	29	8(4)	9*	46,XX (1)	Diploid - aneuploid mosaic (>38%)
					45,XX,-15 (2)	
					47,XX,+15 (1)	
					44,XX,-15,-22 (1)	
					46,XX,dup(15q11.1-q22.2) (3)	
					No result (1)	
10	E	29	7(4)	7	46,XY (2)	Diploid - aneuploid mosaic (>38%)
					46,XY,dup(10q21.1-qter) (1)	
					46,XY,del10q (1)	
					47,XY,+15,del(10q21.3-qter) (1)	
					42,XY,-13,-15,-18,-19 (1)	
					50,XY,+13,+15,+18,+19 (1)	
11	E	29	8(2)	10*	46,XY (9)	Normal
					No result (1)	
12	E	29	7(1)	7	46,XX (4)	Diploid - aneuploid mosaic (<38%)
					44,XX,-11,-20 (2)	
					No result (1)	

*Biopsied blastomeres cleaved before cells were placed in tubes.

### Day 3 embryo mosaicism

A total of 97 blastomeres were collected, and conclusive results were obtained from 92.8% (90/97). No results were obtained from 7 blastomeres due to failure in whole genome amplification. No contamination was found in all of the negative controls. Of the 90 analysed blastomeres, 47.8% (43/90) were euploid with no segmental aberrations. There were similar percentages of blastomeres with single monosomy (11.1%, 10/90), single trisomy (10.0%, 9/90), two aneuploid chromosomes (6.7%, 6/90) and complex abnormalities with 3 or more aneuploid chromosomes (8.9%, 8/90). Segmental changes were noted in 16.7% (15/90) of the blastomeres analysed.

The studied embryos were classified into five categories after aCGH analysis according to the extent and type of chromosomal abnormalities (Table 1). Normal embryos referred to those with normal chromosome number in all of their blastomeres analysed. Diploid-aneuploidy mosaic embryos were those containing both euploid and aneuploid blastomeres. They were sub-divided according to the proportion of aneuploid blastomeres (<38% or > =38%). A cut-off of 38% was used because cryopreserved 8-celled embryos with up to three lysed blastomeres (3/8, 37.5%) after thawing can produce live births [17]. Aneuploid mosaic embryos referred to those containing a mixture of blastomeres with different types of chromosomal abnormalities, while all of the blastomeres in aneuploid embryos had the same type of abnormality. There were 16.7% (2/12) normal embryos, 25% (3/12) diploid/aneuploidy mosaic embryos with <38% abnormal blastomeres, 33.3% (4/12) diploid/aneuploidy mosaic embryos with > =38% abnormal blastomere, 25% (3/12) aneuploid mosaic embryos and 0% (0/12) aneuploid embryo.

### Cell lineage analyses

**Figure 1:**
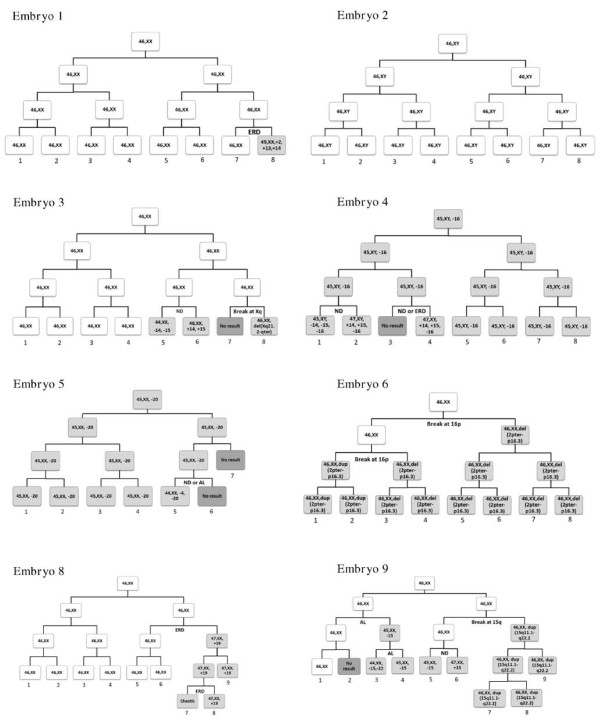
**The reconstructed chromosomal status of blastomeres in embryos 1–6 and 8–9.** The reconstruction of cell lineage is based on results of aCGH and microsatellite marker analysis. Abnormal blastomeres are shown in pale grey and those with no result are shown in dark grey. The reconstruction assumes 1) anaphase lagging (AL), non-disjunction (ND), selective endoreduplication (ERD) or chromosome breakage cause the observed chromosomal aberrations; 2) a minimal number of the above events that could explain the chromosomal status of the embryos; and 3) one event per cell division as far as possible.

**Figure 2:**
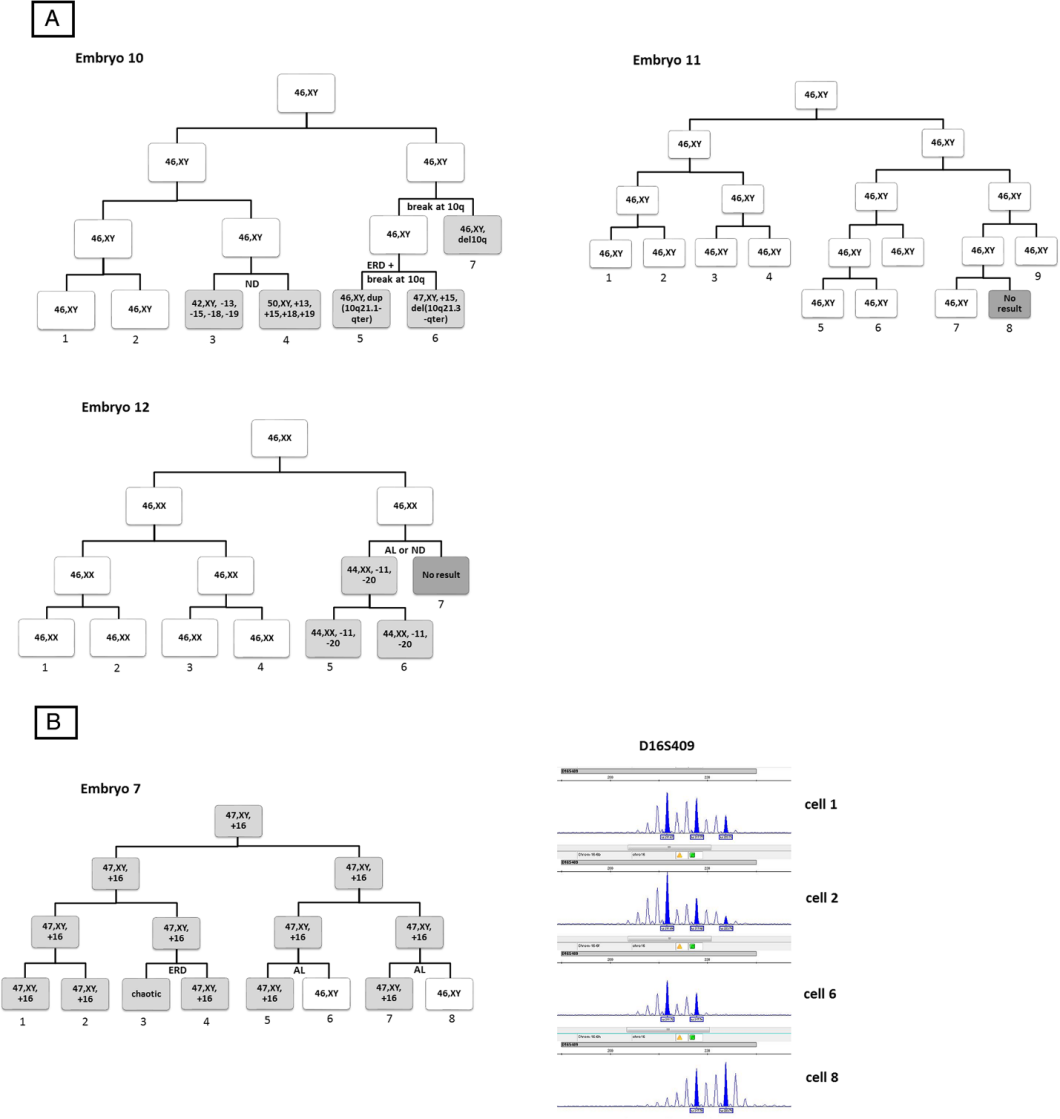
**The reconstructed chromosomal status of blastomeres in embryos 10–12 and embryo 7. A)** Embryos 10–12. The reconstruction of cell lineage is based on results of aCGH and microsatellite marker analysis. Abnormal blastomeres are shown in pale grey and those with no result are shown in dark grey. The reconstruction assumes 1) anaphase lagging (AL), non-disjunction (ND), selective endoreduplication (ERD) or chromosome breakage cause the observed chromosomal aberrations; 2) a minimal number of the above events that could explain the chromosomal status of the embryos; and 3) one event per cell division as far as possible. **B)** Embryo 7 with microsatellite marker results at D16S409. The results from cells 4, 5 and 7 are similar to cells 1 and 2, with three alleles detected at locus D16S409. Therefore, the trisomy is the result of meiotic error. Disomic cells 6 and 8 showed a different set of allele pairs and are probably derived from two independent anaphase lagging events.

All of the studied embryos were graded every 24 h after fertilization, and no trichotomic mitosis was observed. Based on the chromosomal content determined by aCGH, we reconstructed the chromosomal content of the blastomeres in each division. The cell lineages of each embryo are shown in Figure 1, Figure 2A and B. The cell lineages reconstructed were confirmed by analysis of the microsatellite markers on the aneuploid chromosomes (see Additional file 1: Table S1 and Additional file 2: Table S2). For instance, Embryo 7 was derived from an aneuploid zygote with trisomy 16. The meiotic origin of the defect was confirmed by microsatellite marker analysis, which showed that the 5 blastomeres with trisomy 16 had three alleles on the marker D16S409. The 2 normal blastomeres of the embryo showed 2 different combinations of allele pairs, suggesting that they were resulted from two independent AL events.

Out of the 12 embryos, only 3 (25%) embryos (embryos 4, 5 and 7) showed meiotic errors. Mitotic errors occurred in 10 embryos (83.3%, 10/12) and were the main cause of embryo mosaicism in this cohort of embryos. The 12 studied embryos underwent 85 mitotic divisions in the 3 days of culture. There were 16 mitotic errors (18.8%, 16/85) leading to whole chromosome imbalance. The frequencies of endoreduplication (31.3%, 5/16), non-disjunction (25%, 4/16) and anaphase lagging (25%, 4/16) were similar (Table 2). Chromosome breakages occurred in 6 divisions (7.1%, 6/85);

**Table 2:** **Prevalence of mitotic errors on day 3 embryos**

Mitotic error	No. of event	Frequency per mitotic error	Frequency per cell division
ERD	5	31.3%	5.9%
ND	4	25%	4.7%
AL	4	25%	4.7%
ND/ERD	1	6.2%	1.2%
ND/AL	2	12.5%	2.4%
**Total**	**16**		

ERD: endoreduplication; ND: non-dysjunction; AL; anaphase lagging.

## Discussion

This is the first report on the extent of mosaicism in high-quality embryos donated from young women, and more than half of the embryos were from successful assisted reproduction treatment cycles. Previous studies on embryo mosaicism typically used genetically abnormal embryos acquired from the PGD/PGS program [7, 8, 12] or cryopreserved embryos with lysis of some blastomeres after thawing [9]. The average number of cells per day 3 embryo analysed in these studies ranged from 5.2 to 6.9 [8–10, 13]. There is only one study on two chromosomally abnormal embryos from PGD with an average of 8 cells per embryo [7]. In our study, the embryos were donated from patients with a mean age of 30. All of the studied embryos had no blastomere lysis after thawing and developed past the 6-cell stage with good morphology after 24 h of culture. The average number of blastomeres per embryo on day 3 was 7.8. The strength of this study is that we had conclusive result on over 90% of the studied blastomeres. Thus, our data reflect a more complete picture of embryo mosaicism in high quality embryos.

A recent study reported mosaicism in 14 embryos from 9 young couples (mean maternal age: 31.3) with live birth from the same assisted reproduction treatment cycle [10]. In the study, conclusive results could not be obtained in 33.3% (35/105) of the blastomeres due to loss of some blastomeres during thawing or disaggregation and failure of analysis, leading to no result. The low rate of obtaining conclusive results may be partly because the embryos were allowed to succumb overnight, leading to degradation of DNA and subsequent difficulties in analyses. Similarly, only 52% of blastomeres can be analysed in a cohort of embryos diagnosed to be abnormal in the PGD program [8]. In the present study, inconclusive results were found in only 7 blastomeres, and the chromosomal content in 92.8% of the blastomeres was successfully determined.

Among the 12 studied embryos, 16.7% were diploid and 58.3% were diploid-aneuploid mosaic. These percentages are similar to previous studies on surplus embryos. Both Wells and co-workers [13] and Voullaire and co-workers [9] reported a diploid rate of 25% and a diploid-aneuploid mosaic rate of 41.7%. Similar rates were found in good quality embryos from advanced aged women [14] (diploid: 23%, diploid-aneuploid: 46.2%) and young women [10] (diploid: 28.6%, diploid-aneuploid: 57.1%).

If assuming that only diploid embryos could implant, the percentage of diploid embryos in our study is 16.7%, which is much lower than the implantation rate in the frozen-thawed embryo cycle for young women, which is 30.9% in our program. The observation suggests that some of the embryos with a minor proportion of abnormal blastomeres may implant. For instance, only 1 out of 8 blastomeres was abnormal in Embryo 1. It proposed that the implantation potential of mosaic embryos depended on the number of chromosomally abnormal blastomeres in the embryos [18]. Frozen-thawed embryo transfer data show that 8-celled embryos that have three blastomeres (37.5%, 3/8) lysed after thawing are able to develop normally to term [17]. Therefore, it is reasonable to assume that the embryos with less than 38% chromosomally abnormal blastomeres could implant. Among the 7 diploid-aneuploid mosaic embryos, 3 had <38% of abnormal blastomeres. Thus the percentage of studied embryos with implantation potential, i.e., diploid and diploid-aneuploidy mosaic with <38% abnormal blastomeres, is 41.7% (5/12), which is consistent with the implantation rate of frozen-thawed embryos.

The fate of chromosomally abnormal blastomeres is not fully understood. Studies show that the proportion of these blastomeres drops as the embryos develop to the blastocyst stage [19, 20]. Several possibilities may explain the phenomenon. First, a high rate of mosaicism in early cleavage embryos may be due to degradation of the maternal transcripts leading to inadequacy of in cell cycle control and incomplete activation of embryonic genome [21]. The development of cell cycling genes after embryonic genome activation at the 8-celled stage reduces the proportion of abnormal cells formed in later developmental stages. Second, there could be preferential growth of the euploid cells, loss of the aneuploid cells due to apoptosis or reduced division of the abnormal blastomeres [22]. Third, it is possible that some of the abnormal blastomeres undergo “self-correction”. Several mechanisms of self-correction have been proposed including anaphase-lagging or non-disjunction [23–25]. Mertzanidou and co-workers [11] suggested that self-correction mechanisms start after day 4 of preimplantation development. It was once suggested that preferential allocation of abnormal cells to the trophectoderm could be one of the mechanisms, but this was contradicted by later observations [26].

In this study, 48.9% of the blastomeres were euploid with no segmental aberration. The percentage of normal blastomeres is comparable to that in previous reports on day 3 embryos [9, 13] that range from 41% to 56%. We found similar percentages of blastomeres with single monosomy (11.4%) and single trisomy (10.2%). These values vary among other studies. While Voullaire and co-workers [9] reported 27% single monosomy and 3% single trisomy, the corresponding values by Wells and Delhanty [13] are 8% and 17%, respectively. Factors affecting the proportion of monosomy and trisomy are not fully known. A recent retrospective analysis of over 15,000 trophectoderm biopsies showed equal prevalence of trisomies and monosomies [27]. The percentage of blastomeres with more than one aneuploidy in the present study is 15.9%, which is similar to other reports [9, 13].

As over 90% of the blastomeres had a definitive analysis, we reconstructed the cell lineages of each studied embryo and deduced the genotype of their zygotes based on chromosomal content and microsatellite marker analysis of blastomeres on day 3. Among the 12 studied embryos, only 3 chromosomal errors were found at the zygote stage. These chromosome errors could be due to meiotic errors of paternal and/or maternal origins. Although spermatozoa from men with severe oligoasthenoteratozoospermia have increased aneuploidy rates [28], the estimated aneuploidy rate is less than 5% [29, 30], which is much lower than the reported aneuploidy rate of the oocytes (22–57.1%) [31]. Thus the observed errors are likely due to meiotic errors that occurred during oogenesis. Maternal meiotic error is well known to be positively correlated with advanced maternal age [32]. Our studied embryos were donated from young patients. Therefore, a low incidence of meiotic error was expected. Aneuploidy rates of 3–17.9% based on CGH of polar bodies have been reported for young women [31, 33].

In contrast to meiotic error, the rate of mitotic errors does not increase with maternal age [32, 34]. We found 16 mitotic errors in this study resulting in whole chromosome gain or loss. There were 5 endoreduplication events, accounting for 31.3% of the mitotic errors observed (Table 2). Trophoblast cells derived from the trophectoderm of blastocysts undergo physiological endoreduplication to become the polyploid syncytiotrophoblast [35]. Although endoreduplication usually involves the whole chromosome set, selective endoreduplication of isolated chromosomes has been reported in a human tripronucleated zygote [36] and in cleavage stage embryos [11].

Most of the previous studies on the mechanism of aneuploidy in preimplantation embryos were performed by FISH based on a limited number of chromosomes [15]. Ioannou and co-workers [37] studied all 24 chromosomes in blastocysts by 4 rounds of FISH, and concluded that anaphase lagging was the most common mechanism causing post-zygotic abnormalities. However, as only one probe per chromosome and blastocysts diagnosed to be abnormal after PGS were used in the study, the mechanisms of aneuploidy in unselected good quality embryos are not known. We found 4 non-disjunction and 4 anaphase lagging events out of 85 divisions in the studied embryos. In a similar reconstruction analysis on 13 day 4 embryos, 5 non-disjunction and 7 anaphase lagging events were postulated on day 3, but the incidence of non-disjunction increased dramatically as the embryos developed to day 4 [11]. Whether the mechanisms of aneuploidy change with the development of the embryos awaits further investigation. It is noteworthy that there are other possible mechanisms of mitotic error such as premature cell division, chromosome demolition, cell fusion and errors in cytokinesis.

Chromosomal structural aberrations are common in preimplantation embryos, though their true frequency and biological significance are not fully known [8]. Evidence suggests that these aberrations are independent of maternal age [32]. It has been suggested that the prevalence of segmental aberration is higher in frozen-thawed embryos than in fresh embryos [11]. Review of the literature shows a segmental aberration rate per blastomere from 6.3% [13] to 8% [11] in fresh embryos and 7.1% [10] to 12% [11, 14] in frozen-thawed embryos. In the present study, segmental change of chromosome was noted in 17% of the blastomeres after thawing. If there is a difference in the rate of segmental aberration, the difference is likely to be small and is of doubtful significance. It should be noted that the frequency of structural aberrations depends on the resolution of the microarray. Thus, Vanneste and co-workers [8] reported a much higher frequency (70%) with the use of a SNP array having a resolution hundreds-fold higher than that used in the present study.

## Conclusions

In conclusion, a high degree of mosaicism occurred in good-quality embryos from young patients. Less than 20% of the embryos were euploid, and it is likely that some mosaic embryos with a low number of abnormal blastomeres could also implant. In contrast to previous reports, mitotic errors, rather than meiotic errors, were the main cause of mosaicism in this cohort of embryos. Selective endoreduplication, non-disjunction and anaphase lagging contribute similarly to the chromosomal abnormality observed. The weakness of this study is the small number of embryos examined, and the conclusions should be confirmed with a larger study. The high level of mosaicism in day 3 embryos may lead to false positive or false negative results in the PGS cycle. As the level of mosaicism declines with preimplantation development, biopsy on day 5 should reduce the chance of errors due to mosaicism in PGS [37–39].

## Electronic supplementary material

Additional file 1: Table S1: Microsatellite marker analysis of blastomeres from embryos 1, 3 and 4. (PDF 36 KB)

Additional file 2: Table S2: Microsatellite marker analysis of blastomeres from embryos 5, 8, 9, 10 and 12. (PDF 130 KB)

## Abbreviations

aCGH: Array based comparative genomic hybridization
AL: Anaphase lagging
ERD: Endoreduplication
FISH: Fluorescence in situ hybridization
IVF: In vitro fertilization
ND: Non-dysjunction
PCR: Polymerase chain reaction
PGD: Preimplantation genetic diagnosis
PGS: Preimplantation genetic screening
UPD: Uniparental disomy.
