# Supplementary material for: Array comparative genomic hybridization analyses of all blastomeres of a cohort of embryos from young IVF patients revealed significant contribution of mitotic errors to embryo mosaicism at the cleavage stage
Source: Reprod Biol Endocrinol. 2014 Nov 24;12:105. doi: 10.1186/1477-7827-12-105 (PMC4256731; doi:10.1186/1477-7827-12-105)
Supplement: Supplementary file 1 — Additional file 1: Table S1: Microsatellite marker analysis of blastomeres from embryos 1, 3 and 4. (PDF 36 KB) [file 12958_2014_1282_MOESM1_ESM.pdf]

Supplemental Table S1 – Microsatellite marker analysis of blastomeres from embryos 1, 3 and 4

Cell	1	2	3	4	5	6	7	8
**Embryo 1**								
Chromosome 2								
**D2S360**	-/304	289/304	289/304	289/304	289/304	-/289	289/304	289/304
**D2S2300**	-/434	421/434	421/434	421/434	421/434	421/434	-/434	421/434
**STR 2-02**	327/335	327/335	327/-	327/-	327/335	-/335	327/335	327/335
Chromosome 14								
**STR 14-02**	193/197	193/197	193/197	193/197	193/197	193/197	193/197	193/-
**D14S1016**	264/279	264/279	264/279	264/279	264/279	264/279	264/279	264/279
**Embryo 3**								
Chromosome 14								
**D14S1016**	272/279	272/279	272/279	272/279	279	*	-	272/279
Chromosome 15								
**D15S161**	389/397	389/397	389/397	289/397	397	*	-	389/397
**D15S978**	183/189	183/189	183/-	183/189	183	*	-	183/189
**Embryo 4**								
Chromosome 14								
**STR 14-02**	180	180/193	-	180/193	180/193	180/193	180/193	180/193
Chromosome 15								
**D15S161**	397	389/397	-	389/397	389/397	389/397	389/397	389/397
**D15S978**	-	183/189	-	-/189	183/189	183/189	183/189	183/189
Chromosome 16								
**D16S409**	-	212	-	212	212	-	212	212
**D16S3099**	265	265	-	265	265	265	265	265

Blastomeres with aneuploid chromosome are shown in the shaded blocks. Only informative markers with distinctively separated alleles were included. Only abnormal embryos are shown.

*sample lost after aCGH, no microsatellite marker analysis was performed
