# Supplementary material for: Array comparative genomic hybridization analyses of all blastomeres of a cohort of embryos from young IVF patients revealed significant contribution of mitotic errors to embryo mosaicism at the cleavage stage
Source: Reprod Biol Endocrinol. 2014 Nov 24;12:105. doi: 10.1186/1477-7827-12-105 (PMC4256731; doi:10.1186/1477-7827-12-105)
Supplement: Supplementary file 2 — Additional file 2: Table S2: Microsatellite marker analysis of blastomeres from embryos 5, 8, 9, 10 and 12. (PDF 130 KB) [file 12958_2014_1282_MOESM2_ESM.pdf]

Supplemental Table S2 - Microsatellite marker analysis of blastomeres from embryos 5, 8, 9, 10 and 12

Cell	1	2	3	4	5	6	7	8	9
**Embryo 5**								NA	NA
Chromosome 20									
**D20S186**	306	306	306	306	-	-	-		
**D20S109**	253	253	253	253	-	-	-		
**D20S884**	308	308	308	308	-	-	-		
**D20S888**	354	354	354	354	-	-	-		
**Embryo 8**									
Chromosome 19									
**D19S112**	188/202	188/202	188/202	188/202	188/202	188/202	188/-	188/202	188/202
**D19S412**	250/257	250/257	250/257	250/257	250/257	250/257	250/-	250/257	257/-
**Embryo 9**									
Chromosome 15									
**D15S161**	385/391	-	391	391	391	385/391	385/391	-/391	*
**D15S978**	185/202	-	185	185	185	-	185/202	185/202	*
**Embryo 10**									
Chromosome 15								NA	NA
**D15S978**	183/200	183/200	200	183/200	183/200	183/200	183/200		
**Embryo 12**									
Chromosome 11								NA	NA
**D11S937**	268/276	268/270	268/276	268/276	276	276	-		
Chromosome 20									
**D20S186**	305/315	306/315	306/315	306/315	315	315	-		
**D20S109**	233/255	233/255	233/255	233/255	255	255	-		

Blastomere with aneuploidy chromosome were shown in shaded block. Only informative markers with distinctively separated alleles were included. Only abnormal embryo were shown.

*sample lost after aCGH, no microsatellite marker analysis was performed

NA- not applicable
